# New Medical School—Progress

**Published:** 1850-07

**Authors:** 


### ARTICLE II.

### NEW MEDICAL SCHOOL—PROGRESS.

We have received the *first Annual Announcement* of the Faculty of the Medical Department of the University of Michigan, located at Ann Arbor. Although one of the youngest States in the Union, Michigan deserves, if she does not enjoy, the credit of having adopted a more complete and liberal system of education than is to be found in any other State or country of which we have any knowledge. Having received from Congress a rich donation of public lands for school purposes, she did not follow the example of some of her sister states of the West, and allow half their value to be wasted by negligence and bad management, but faithfully devoted them to the establishment of a complete system of schools for the whole State; embracing primary, or district schools, sectional, or academical institutions, and a State University, which is provided with Departments of Law and Medicine. We are glad to see by the circular before us that the Regents of the University, in organizing the Medical Department have been actuated by the most liberal and enlightened motives.

The following extracts will show both the actual regulations adopted, and some of the objects they were designed to accomplish:—

"Prominent among the proposed innovations, are *pre-requisitions* to entrance upon the study of medicine—extension of the lecture terms—thorough daily examinations upon the topics discussed in the lectures—and proofs of a higher degree of proficiency, prior to granting the diploma. The exigencies of the time, it is believed, call for the establishment of an institution in which these ideas can be carried into practice; and no school, perhaps, is better situated for the realization of the objects proposed, than the one referred to in this Announcement.

"Fortunately, Michigan has the desire and the ability to possess herself of the honor of being the pioneer in this important improvement. Her statutes require that the University "provide the inhabitants of this State with the means of acquiring a thorough knowledge of the various branches of Literature, Science, and the Arts," the "Department of Medicine and Surgery" being especially designated. The law provides that the instruction given in this Department of the University, as in all others, shall be gratuitous, the Professors being paid from the munificent fund provided the State of Michigan, for this purpose, by the general Government."

The following are among the regulations adopted, viz:---

"Every candidate for admission shall present to the Faculty satisfactory evidence of good moral character, and also of such literary attainments as have been recommended by the National Medical Association, viz: A good English education; the knowledge of Natural Philosophy, the elementary Mathematical Sciences, and such an acquaintance with the Latin and Greek languages as will enable the student to appreciate the technical language of medicine, and read and write prescriptions.

"The Course of Lectures in this Department will commence on the first Wednesday in October and continue until the third Wednesday in April.

"There will be *four* lectures daily, except on Saturday, which day will be devoted to clinical instruction and reading of Thesis.

"Each candidate for graduation shall so announce himself at the close of his first, or the commencement of his second course, and shall be examined upon the subjects of Anatomy, Physiology, Materia Medica, and Chemistry.

In order that any student may be admitted to the Degree of Doctor of Medicine, he shall exhibit evidence of having pursued the study of medicine and surgery for the term of three years, with some respectable practitioner of medicine (including lecture terms); must have attended two full courses of Lectures, the last of which must have been in the Medical Department of the University of Michignn—must be twenty-one years of age—must have submitted to the Faculty an original Thesis, in his own hand-writing, on some medical subject; and have passed an examination, held at the close of the term, satisfactory to the Faculty.

"A charge of *ten dollars, paid once*, is the only fee required of the student. A deduction of one year is made from the term of study in favor of those who have graduated in the

Literary Department of the University, or in other respectable colleges. And clergymen, members of the legal profession, and graduates of other respectable medical institutions, may be permitted to attend as "honorary members of the Medical Department."

We notice only two serious obstacles to the complete success of these judicious regulations. One is, the location of the school where no adequate *Clinical*, or *Hospital* advantages can ever be provided; and the other, beginning with only five members of the Faculty. Still much credit is due to the Board of Regents for the adoption of such a liberal system, and especially to our friend Dr. Pitcher, of Detroit, one of its most influential members. N. S. D.

